# Liver Stiffness Is Associated with the Burden of Carotid and Systemic Atherosclerosis in an Unorganized Cohort of Patients 40–64 Years Old

**DOI:** 10.3390/diagnostics12102336

**Published:** 2022-09-27

**Authors:** Alla Kuznetsova, Anastasiya Dolgushina, Albina Savochkina, Lubov Pykhova, Veronika Sumerkina, Anna Selyanina, Yana Kudrinskaya, Vadim Genkel

**Affiliations:** 1 Department of Hosptital Therapy, South-Ural State Medical University, 454092 Chelyabinsk, Russia; 2 Central Research Laboratory, South-Ural State Medical University, 454092 Chelyabinsk, Russia; 3 Department of Internal Medicine, South-Ural State Medical University, 454092 Chelyabinsk, Russia

**Keywords:** liver fibrosis, atherosclerosis, plaque burden, liver stiffness, transient elastometry

## Abstract

Background: The aim of the study is to research the relationship between the severity of liver fibrosis and the burden of carotid and systemic atherosclerosis. Methods: The study includes 163 patients 40 to 64 years of age without atherosclerotic CVD or liver disease. All patients underwent duplex scanning of the carotid and lower limb arteries. All patients underwent transient liver elastometry using the FibroScan (Echosens, France). Results: Carotid plaque was detected in 110 (67.5%) patients. Based on the results of linear regression analysis, relationships between liver stiffness and carotid total plaque area (r = 0.21; *p* = 0.025) were found. Significant relationships were established between liver stiffness and atherosclerosis burden score based on the results of linear regression (r = 0.17; *p* = 0.029). Liver stiffness showed moderate diagnostic performance (AUC 0.666; *p* = 0.01) with regard to generalized atherosclerosis. An increase in liver stiffness >4.5 kPa was associated with an odds ratio of generalized atherosclerosis of 3.48 (95% CI 1.07–11.3; *p* = 0.038) after adjusting confounding factors. Conclusion: Among patients 40–64 years of age without established atherosclerotic CVD and liver disease, liver stiffness directly correlates with the burden of carotid and systemic atherosclerosis. Liver stiffness showed moderate diagnostic performance (AUC 0.666; *p* = 0.01) with regard to generalized atherosclerosis.

## 1. Introduction

Affecting 25% of the adult population worldwide, non-alcoholic fatty liver disease (NAFLD), or metabolic-associated fatty liver disease (MAFLD), is currently the most common liver disease [1]. At the same time, the frequency of new cases of NAFLD is steadily increasing. Thus, according to a cohort study conducted in the United States, within the period from 1997 to 2014, the incidence of new cases of NAFLD increased by 5 times or from 62 per 100,000 patient-years to 329 per 100,000 patient-years [2]. In turn, the growing global burden of NAFLD determines, most probably with a certain level of time lag, a measurable growing burden of cardiovascular disease (CVD). In one of the latest meta-analyses by L. Alon et al., it was found that the presence of NAFLD is associated with an increase in the relative risk (RR) of myocardial infarction by 1.66 times (95% confidence interval (CI) 1.39–1.99), ischemic stroke by 1.41 times (95% CI 1.29–1.55) and heart failure by 1.62 times (95% CI 1.43–1.84) [3]. According to the analysis of the US National Vital Statistics System, CVD is the second most common cause of death in patients with NAFLD (the first place was occupied by liver-related causes) [4]. Although NAFLD is now considered a global challenge for healthcare systems and societies as a whole, measures taken in preparation for dealing with it are clearly unsatisfactory and there are in fact no national strategies present worldwide to control NAFLD [5,6]. The adaptation and implementation of preventive efforts to counter cardiovascular diseases and early diagnosis of atherosclerosis into the treatment algorithms and methods used to treat patients with NAFLD should become an integral part of care strategies [7,8,9].

At all stages of the NAFLD continuum, fibrogenesis processes in the liver are present [10]. In most cases, the progression of fibrosis in NAFLD develops relatively slowly, increasing by an average of 1 METAVIR stage over 14.3 years (in NASH, over 7.1 years) [11]. Despite that significant levels of liver fibrosis are observed in no more than 7.5% to 15% of patients with NAFLD, it is assumed that it is in fact the severity of liver fibrosis that can serve as an indicator of a patient’s risk level of suffering hepatic and extrahepatic manifestations of NAFLD, including atherosclerotic CVD [12,13,14]. In a meta-analysis of 36 prospective studies, which included more than 5.8 million participants, the risk of fatal or nonfatal cardiovascular events significantly increased in accordance with the increasing severity of fibrosis [15]. A series of more recent studies have also confirmed the dose-dependent effects of liver fibrosis on the development of cardiovascular and other extrahepatic adverse events [16,17]. Thus, it is perhaps the assessment of liver fibrosis that may make it the most important tool in the assessment of cardiovascular risks in patients with NAFLD and other categories of patients. Using a random sampling of middle-aged patients, the aim of this study was to research the relationship between the severity of liver fibrosis on the one hand, and the burden of carotid and systemic atherosclerosis on the other.

## 2. Materials and Methods

The study included outpatients attending for a scheduled periodic medical examination at Chelyabinsk City Clinical Hospital No. 1. Inclusion criteria for the study were as follows: age from 40 to 64 years; patient’s consent to participate in the study; and absence of exclusion criteria. The target population represented working-age patients eligible for systematic cardiovascular screening. The exclusion criteria were as follows: previously established atherosclerotic CVD (a history of cerebrovascular disease, the presence of coronary heart disease, peripheral arterial disease and/or the revascularization of the coronary or peripheral arteries); established liver disease; severe dysfunction of the liver and kidneys (a decrease in the glomerular filtration rate of less than 30 mL/min/1.73 m^2^); and malignant neoplasms and established chronic inflammatory diseases.

All patients signed an informed consent form upon inclusion in the study. The study’s protocol was approved by the ethics committee of the South Ural State Medical University (protocol No. 10 dated 27 October 2018).

### 2.1. Duplex Scanning of the Carotid and Lower Limb Arteries

All the patients in the study underwent duplex scanning of the carotid and lower limb arteries. The following vessels were examined from both sides in longitudinal and transverse sections along their entire length: the common carotid arteries (CCA) with CCA bifurcation, the internal carotid arteries (ICA), the external carotid arteries (ECA), the common femoral arteries (CFA), the superficial femoral arteries (SFA), the popliteal arteries (PA) and finally the arteries of the tibial segment. The study was carried out in B-mode, color mapping mode and pulsed Doppler and also with a linear transducer at a frequency of 10 MHz on a Canon Aplio 400 (Tokyo, Japan) digital ultrasonic multifunctional diagnostic scanner.

Atherosclerotic plaque was considered as a focal thickening of the intima-media complex of more than 1.5 mm or 0.5 mm more than the surrounding intima-media thickness, or 50% more than the intima-media thickness of the adjacent areas of the CCA [18]. The percentage of stenosis was measured planimetrically in B-mode according to the diameter in the cross-section of the vessel with the percentage of stenosis being determined according to the European Carotid Surgery Trial (ECST) method [19]. The carotid total plaque area (cTPA) was used as an indicator of the carotid plaque burden using the measurement technique described earlier [20,21]. The burden of systemic atherosclerosis was assessed using the ultrasound atherosclerosis burden score (ABS) [22]. The total score on the ABS scale was calculated based on the results of an assessment of the presence of plaque in the bifurcations of the CCA and CFA on both sides. If at least one plaque was found in one of the studied areas one point was awarded. Thus, the score on the ABS could possess values from 0 to 4. All vascular ultrasound examinations were performed by a single trained operator. According to the intraobserver variability assessment of the cTPA measurement, good reproducibility was established (intraclass correlation coefficient 0.992 (95% CI 0.969–0.998). The operator was blinded to the liver elastometry results.

### 2.2. Transient Liver Elastometry and Liver Ultrasound

All patients underwent transient liver elastometry using the FibroScan device (Echosens, Paris, France). The choice of sensor (M or XL) was made depending on the skin–liver capsule distance [23]. Liver stiffness values were determined using the median result of at least 10 valid measurements with an interquartile range < 30%. All studies were performed by a single trained and qualified operator. All examinations were performed by a single independent trained operator. The operator was blinded to the duplex scanning results.

All patients underwent a transabdominal ultrasound examination of the liver carried out with a convex probe at a frequency of 3.5 MHz using a Canon Aplio 400 (Tokyo, Japan) digital ultrasound multifunctional diagnostic scanner. The semiquantitative determination of the severity of liver steatosis was performed using the Hamaguchi scale [24].

### 2.3. Laboratory Examination

The following biochemical laboratory blood parameters were obtained after fasting for at least 8 h: total cholesterol, low-density lipoprotein cholesterol, high-density lipoprotein cholesterol, triglycerides, glycated hemoglobin and creatinine with subsequent estimated glomerular filtration rate (eGFR) calculation according to the CKD-EPI formula.

### 2.4. Statistical Analysis

The data that were obtained were analyzed using the statistical data analysis package MedCalc (ver. 20.019, MedCalc Software Ltd., Osten, Belgium) and IBM SPSS Statistics (ver. 18, SPSS Inc., Chicago, IL, USA). Qualitative variables were described by absolute and relative frequencies (percentages). Quantitative variables were described by the median (Me) indicating the interquartile interval [25th percentile and 75th percentile]. Spearman’s correlation analysis was used to determine the relationship between the indicators. Any significant differences between more than two groups were assessed using the Kruskal–Wallis test followed by a pairwise comparison using the Mann–Whitney test. Cochran–Armitage’s Chi-square test for trend was used to assess the significance of differences in the frequency distribution of nominal variables between more than two groups. Differences were considered statistically significant if they were at a critical significance level of 0.05.

To establish the threshold values of the studied parameters, receiver operating characteristics (ROC) analysis was performed to obtain the determination of sensitivity, specificity, PPV and NPV. The calculation of the area under the characteristic curve (AUC) with a 95% confidence interval (CI) and Youden index were also carried out.

## 3. Results

The study included 163 patients with a median age of 49.0 years of age. Table 1 shows the detailed clinical characteristics of the patients.

**Figure 1:**
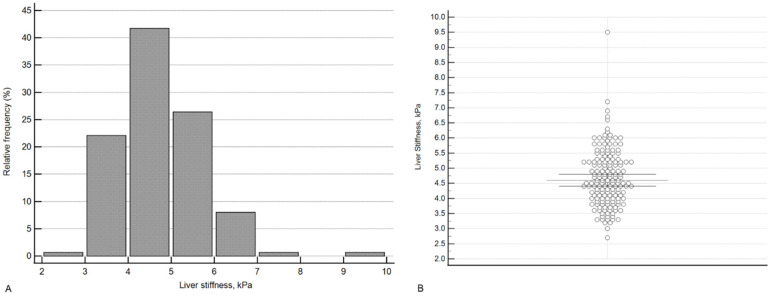
Distribution of liver stiffness values in relative (**A**) and absolute (**B**) values. In Figure 1B, each point represents a single case. The lines indicate the median and the 95% confidence interval for the median.

Figure 1 shows the distribution of liver stiffness values in the study group.

### 3.1. Relationships between Liver Stiffness and the Burden of Carotid Atherosclerosis

**Figure 2:**
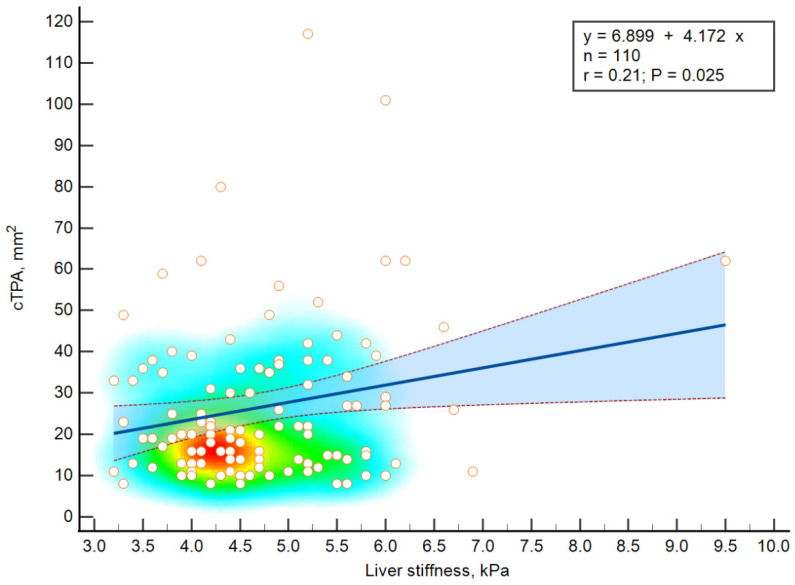
Results of linear regression analysis. The blue line represents the regression line, the dashed lines represent the 95% confidence interval. The heatmap reflects the density of observations.

Carotid plaque was detected in 110 (67.5%) of the patients with median cTPA values of 20.5 mm^2^. Based on the results of linear regression analysis, significant relationships between liver stiffness and cTPA (see Figure 2) were found.

An increase in the liver stiffness value by 1 kPa was associated with an increase in cTPA by 4.17 mm2 (*p* = 0.025). Additionally, patients with liver stiffness values corresponding to the fourth quartile (>5.20 kPa) had significantly higher (*p* = 0.032) maximal carotid stenosis values—25.0% (0.00; 32.2) versus 22.5% (0.00; 28.0).

### 3.2. Relationships between Liver Stiffness and the Systemic Atherosclerosis Burden

**Figure 3:**
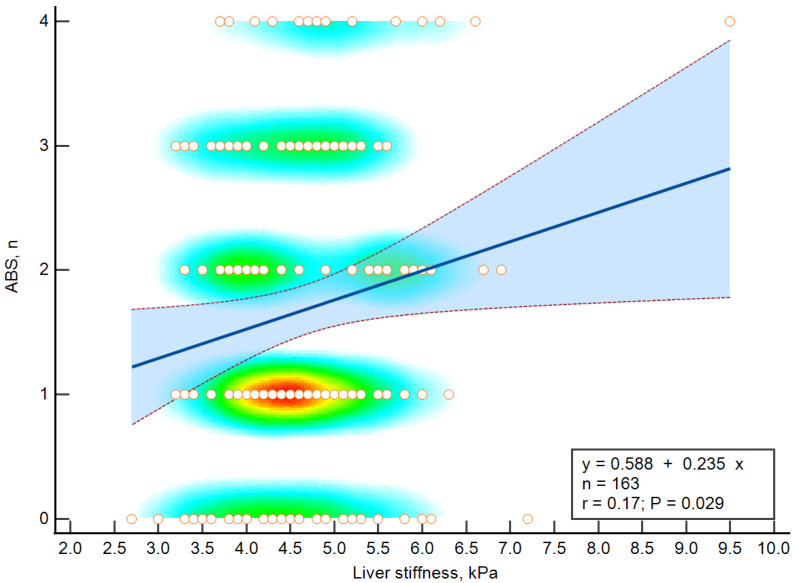
Relationships of liver stiffness and ABS. The blue line represents the regression line, the dashed lines represent the 95% confidence interval. The heatmap reflects the density of observations.

Significant relationships were established between liver stiffness and ABS based on the results of linear regression analysis (see Figure 3). Table 1 shows the distribution of patients by ABS score.

**Figure 4:**
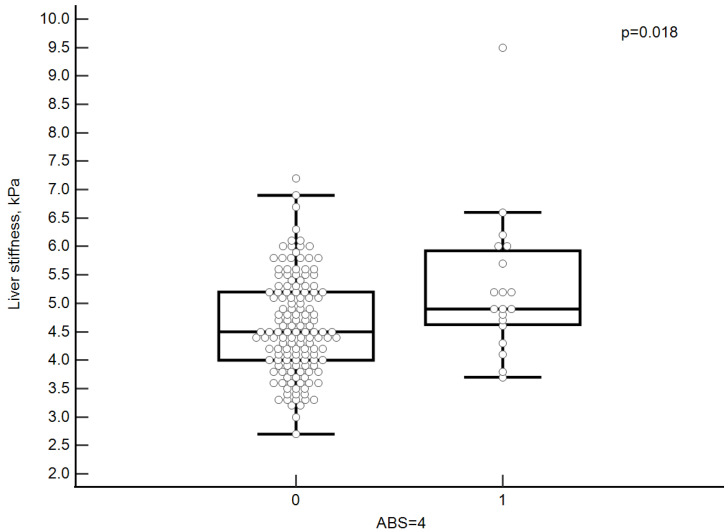
Liver stiffness values in patients with generalized atherosclerosis (ABS = 4). Each point represents a single case. The lines in the figure represent (from inside to outside): median, upper and lower quartile, minimum and maximum.

Patients with generalized atherosclerosis (ABS = 4) had statistically significantly higher liver stiffness values (see Figure 4).

**Figure 5:**
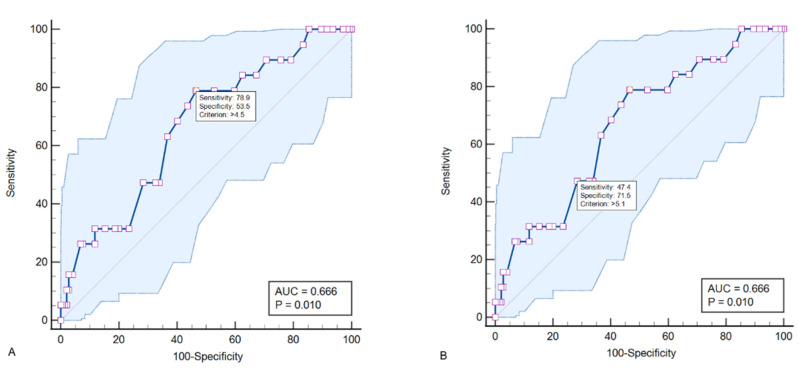
ROC curves demonstrating the diagnostic value of liver stiffness in relation to generalized atherosclerosis. Sub-figures (**A**,**B**) show the selected cut-off points that provide optimal values of sensitivity (**A**) and specificity (**B**).

To assess the potential diagnostic value of liver stiffness in the detection of generalized atherosclerosis (see Figure 5 and Table 2) an in-depth ROC analysis was performed.

An increase in liver stiffness greater than 4.5 kPa was associated with an odds ratio of having generalized atherosclerosis of 3.48 (95% CI 1.07–11.3; *p* = 0.038) after adjusting for sex and age according to the results of logistic regression analysis. However, in the fully adjusted model (age, sex, hypertension, smoking, obesity, TC, LDL-c, TG, eGFR and glucose), the effect of liver fibrosis on the odds ratio of generalized atherosclerosis was not significant (OR 2.73; 95% CI 0.62–11.9; *p* = 0.181).

## 4. Discussion

The severity of liver fibrosis is one of the main determinants of a poor prognosis in patients with liver disease. In a study by P. Angulo et al., it was found that liver fibrosis is a predictor of liver-related events, in contrast to other histological characteristics of NAFLD (steatohepatitis, NAFLD activity index) [25]. These data were not only confirmed by the results of later meta-analyses, but also replicated in relation to extrahepatic adverse events: cardiovascular events, new cases of diabetes mellitus and all-cause death [16,26,27,28,29,30]. Moreover, there is a growing body of evidence that shows, on the one hand, the probable greater than previously believed prevalence of liver fibrosis among the general population, and, on the other hand, the independent prognostic significance of liver fibrosis in connection to cardiovascular events in the general population [31,32]. Programs for population screening for liver fibrosis when discussed in this context show that it is important to develop specific algorithms and protocols for cardiovascular diagnostics and prevention [33].

The main results of the current study are: (1) in an unorganized population of middle-aged patients, liver stiffness values directly correlated with cTPA; (2) as liver stiffness increased, there was an increase in the systemic atherosclerosis burden; and (3) liver stiffness showed moderate diagnostic performance (AUC 0.666; *p* = 0.01) in relation to generalized atherosclerosis.

Several clinical studies previously demonstrated relationships between liver fibrosis and atherosclerosis in various clinical settings. In the work of J.L. Jin et al., an increase in the surrogate serum marker of liver fibrosis FIB-4 was shown to be associated with the presence of coronary calcium, as well as the number of affected coronary arteries and the Gensini index [34]. In a study by C.M. Perdomo et al., liver fibrosis assessed by transient elastography was also associated with the presence of coronary calcium, regardless of confounding factors (abdominal obesity, diabetes mellitus, dyslipidemia, hypertension, prediabetes and obesity) [35]. T. Arai et al. studied the relationship of various histological characteristics of NAFLD and the maximum carotid intima-media thickness and it was found that only fibrosis was significantly associated with maximum intima-media thickness, and not steatosis, inflammation or ballooning [36]. The diagnostic value of serum fibrosis markers (AUC 0.620 to 0.674) in detecting a maximum carotid intima-media thickness ≥ 1.2 mm was comparable to that of histologically assessed liver fibrosis (AUC 0.672). The key importance of our data, among other things, lies in the fact that an increase in the OR of the presence of generalized atherosclerosis was found when there was an increase in liver stiffness within the F0–F1 range. In our opinion, the loss of statistical significance in the fully adjusted model is explained by the shared risk factors and mechanisms of atherosclerosis and liver fibrosis in NAFLD patients. However, this does not diminish the potential diagnostic utility of transient liver elastography in the identification of patients with subclinical atherosclerosis.

The strengths supporting the presented study are the assessment of liver fibrosis by transient elastography and the determination of subclinical atherosclerosis by assessing the burden of carotid and systemic atherosclerosis. It should be noted that surrogate serum markers of liver fibrosis are not optimal screening tools for liver fibrosis in the general patient population, as it has been found that in almost one-third of the cases of increased FIB-4 and NFS, liver fibrosis is not detected [37]. In addition to the aforementioned, serum surrogate markers of liver fibrosis were shown to demonstrate good diagnostic efficiency in patients over 60 years of age who do not have diabetes mellitus and obesity, while their effectiveness in obese patients under 60 years of age is significantly lower [38]. At the same time, it is in the category of patients aged 40 to 60 years of age that the timely diagnosis of NAFLD and liver fibrosis is of particular importance because of the greatest impact of NAFLD on life expectancy in this age group [39]. On the other hand, cTPA and ABS are good and reproducible markers that represent the burden of atherosclerosis and are independently associated with the risk of cardiovascular events, which is only partially applicable, for example, when applied to the intima-media thickness [20,40,41,42].

The general mechanisms present in the development and progression of NAFLD, liver fibrosis and atherosclerosis are all currently known and continue to be studied [43,44]. It is likely that even in the absence of significant liver fibrosis, hepatic stiffness thresholds can be defined in order to represent the cumulative effect of cardiometabolic risk factors. Thus, in our study, patients with liver stiffness values of >4.5 (or >5.1) kPa are likely to be the patients who will benefit most from screening for polyvascular atherosclerosis, subsequent initiation of cardiovascular prevention measures and individualized follow-ups.

The study that has been presented possesses the following limitations: (1) the single center nature of the study, (2) the lack of a quantitative assessment of hepatic steatosis (CAP) and (3) a mixed sample of patients—making it difficult to extrapolate the results to apply them to other categories of patients.

## 5. Conclusions

Among patients 40–64 years of age without established atherosclerotic CVD and liver disease, liver stiffness, which was determined by transient elastography, directly correlates with the burden of carotid and systemic atherosclerosis. Liver stiffness showed moderate diagnostic performance (AUC 0.666; *p* = 0.01) with regard to generalized atherosclerosis.

## Figures and Tables

**Table 1:** Clinical characteristics of patients.

Characteristics	Patients (*n* = 163)
Male, *n* (%)/Female, *n* (%)	77 (47.2)/86 (52.8)
Age, years, Me (LQ; UQ)	49.0 (44.0; 55.0)
BMI, kg/m2, Me (LQ; UQ)	27.5 (23.5; 31.0)
Obesity, *n* (%)	47 (28.8)
Abdominal obesity, *n* (%)	94 (57.7)
Smoking, *n* (%)	34 (20.9)
T2DM, *n* (%)	4 (2.50)
Hypertension, *n* (%)	65 (39.9)
Βeta-blockers, *n* (%)	8 (4.90)
Renin-angiotensin system inhibitors, *n* (%)	35 (21.5)
Diuretics, *n* (%)	10 (6.10)
Statins, *n* (%)	20 (12.3)
TC, mmol/l, Me (LQ; UQ)	5.87 (4.98; 6.59)
LDL-C, mmol/l, Me (LQ; UQ)	3.64 (2.94; 4.52)
HDL-C, mmol/l, Me (LQ; UQ)	1.37 (1.10; 1.61)
TG, mmol/l, Me (LQ; UQ)	1.20 (0.83; 2.01)
Glycated hemoglobin, %, Me (LQ; UQ)	5.57 (5.18; 6.05)
eGFR, mL/min/1.73 m^2^, Me (LQ; UQ)	77.5 (65.0; 90.0)
Carotid plaque, *n* (%)	110 (67.5)
Maximal carotid stenosis, %, Me (LQ; UQ)	24.0 (0.00; 30.0)
cTPA, mm2, Me (LQ; UQ)	20.5 (13.0; 36.0)
Femoral plaque, *n* (%)	74 (45.4)
ABS 0, *n* (%)	35 (21.5)
ABS 1, *n* (%)	46 (28.2)
ABS 2, *n* (%)	36 (22.1)
ABS 3, *n* (%)	27 (16.6)
ABS 4, *n* (%)	19 (11.7)
Liver stiffness, kPa, Me (LQ; UQ)	4.60 (4.00; 5.20)
Liver steatosis, *n* (%)	74 (45.4)

BMI = body mass index; TC = total cholesterol; HDL-C = high-density lipoprotein cholesterol; LDL-C = low-density lipoprotein cholesterol; TG = triglycerides; eGFR= estimated glomerular filtration rate; T2DM = type 2 diabetes mellitus; cTPA—carotid total plaque area; ABS = atherosclerosis burden score; obesity was defined as a BMI ≥30 kg/m^2^; abdominal obesity was defined as a waist circumference of more than 80 cm in women and more than 102 cm in men.

**Table 2:** Results of ROC analysis.

Characteristics	AUC (95% CI)	Cut-Off	Se	Sp	Youden Index	PPV	NPV	*p*
Liver stiffness	0.666 (0.589–0.738)	>4.5	78.9	53.5	0.324	18.3	95.1	0.01
		>5.1	47.4	71.5	0.195	18.0	91.2	

AUC = area under curve; Se = sensitivity; Sp = specificity; PPV = positive predictive value; NPV = negative predictive value.

## Data Availability

The data presented in this study are available on request from the corresponding authors.
